# Massive Unilateral Pyonephrosis with Extreme Leukocytosis in a Cat: A Case Report

**DOI:** 10.3390/ani16162604

**Published:** 2026-08-20

**Authors:** Martyna Małż, Magdalena Morawska, Krystyna Makowska

**Affiliations:** 1 Department of Clinical Diagnostics, Faculty of Veterinary Medicine, University of Warmia and Mazury in Olsztyn, Oczapowskiego 14, 10-957 Olsztyn, Poland; martyna.malz@uwm.edu.pl (M.M.); krystyna.makowska@uwm.edu.pl (K.M.); 2 Department of Surgery and Radiology with Clinic, Faculty of Veterinary Medicine, University of Warmia and Mazury in Olsztyn, Oczapowskiego 14, 10-957 Olsztyn, Poland

**Keywords:** cat, pyonephrosis, leukemoid reaction, SDMA, azotaemia, nephrectomy

## Abstract

Pyonephrosis is a rare, life-threatening suppurative disease of the kidney characterised by the accumulation of purulent exudate within the renal pelvis and the destruction of renal parenchyma. It is typically associated with azotaemia and urinary tract obstruction. Extreme leucocytosis (>100 × 10^3^/µL), known as a leukemoid reaction, has been rarely reported in feline pyonephrosis. A 13-year-old neutered domestic shorthair female cat was brought to a veterinary clinic with symptoms of lethargy and apathy, as well as a significantly enlarged abdominal cavity. Physical examination revealed dehydration and a large, non-painful mass occupying the left abdominal cavity. Haematology revealed severe leucocytosis of 123.22 × 10^3^/µL and anaemia with haematocrit (HCT) 18.6% and serum biochemistry showed elevated SDMA 17 µg/dL, AST 97 U/L, with hypokalaemia, hypochloraemia and hypoproteinaemia. Creatinine (1.5 mg/dL) and blood urea nitrogen (BUN) (23 mg/dL) remained within reference values. Abdominal X-ray and ultrasound revealed a large mass of anechoic structure encompassing the entire left side of the abdominal cavity, consistent with pyonephrosis. Microbiological studies of purulent material obtained by ultrasound-guided fine-needle aspiration and culturing revealed the presence of *Escherichia coli*. The day after admission, the patient underwent surgical laparotomy with unilateral nephrectomy. Histopathology confirmed severe inflammatory infiltration of the mucosa, connective tissue proliferation and fibrosis, with partial glomerular hyalinisation and without cellular atypia. Six days after surgical intervention, the leukocyte count decreased to 44.81 × 10^3^/µL. The patient was alive and clinically healthy 3 months after surgery. This case illustrates that massive unilateral pyonephrosis can occur in geriatric cats without azotaemia due to the compensatory function of the contralateral kidney. Marked leucocytosis of 123.22 × 10^3^/µL probably represents a leukemoid reaction secondary to severe suppurative inflammation. Moreover, pyonephrosis should be considered in the differential diagnosis of large abdominal masses in cats, even in the presence of a normal creatinine level.

## Simple Summary

Pyonephrosis is a serious condition in which a kidney becomes filled with pus because of infection and impaired urine drainage. It can be life-threatening, especially when diagnosis is delayed, because the clinical signs may be vague and may resemble other abdominal diseases. This case report describes a 13-year-old cat that presented with weakness, apathy, and a markedly enlarged abdomen. Diagnostic imaging revealed a very large abdominal lesion originating from the left kidney. The kidney was severely damaged and filled with purulent material, and bacterial culture confirmed infection. Despite the complete destruction of one kidney, routine kidney blood values remained within the normal range, probably because the opposite kidney was still functioning. However, an early kidney marker, symmetric dimethylarginine (SDMA), was increased. The cat also had an extremely high white blood cell count, consistent with a strong inflammatory reaction. Surgical removal of the affected kidney led to clinical improvement, and the cat was healthy three months post-surgery. This case shows that pyonephrosis should be considered in cats with large abdominal masses even when routine kidney values are normal.

## 1. Introduction

Pyonephrosis is a severe, suppurative infection of the renal collecting system characterised by the accumulation of purulent material within a dilated renal pelvis, typically secondary to urinary tract obstruction. In cats, pyonephrosis has most commonly been reported in association with ureteral obstruction, such as ureterolithiasis or strictures, which leads to urine stasis, bacterial proliferation, and progressive destruction of renal parenchyma. However, the underlying cause is not identified in all cases. In the present case, no ureterolith, stricture, or other obstructive lesion was identified at surgery, and the cause of the ureteral thickening remains undetermined. *Escherichia coli* is the most frequently isolated pathogen from purulent material, although *Proteus* spp., *Staphylococcus* spp., and *Klebsiella* spp. have also been reported. Clinically, pyonephrosis represents an advanced and potentially life-threatening stage of upper urinary tract infection [1,2].

In cats, pyonephrosis is rarely documented in the literature, with fewer than 15 case reports published to date. Most reported cases have been associated with ureteral obstruction due to urolithiasis or strictures, and the majority of affected cats presented with azotaemia due to bilateral disease or compromise of the contralateral kidney [3,4]. Clinical signs are often non-specific and may include lethargy, anorexia, vomiting, abdominal distension and dysuria. Diagnosis relies on abdominal imaging, particularly ultrasound and/or X-ray, which typically reveals a distended kidney with thickened wall and echogenic fluid content, and is confirmed by cytology and culture of aspirated material.

Leukemoid reaction, defined as extreme leucocytosis exceeding 50 × 10^3^/µL secondary to severe inflammatory or infectious disease, is uncommon in cats and has been described in association with pyometra, pancreatitis and feline infectious peritonitis [5,6,7]. To the authors’ knowledge, extreme leucocytosis exceeding 100 × 10^3^/µL has not been previously reported in feline pyonephrosis.

Symmetric dimethylarginine (SDMA) is a methylated amino acid that is eliminated exclusively by glomerular filtration. In cats, SDMA increases earlier than creatinine when glomerular filtration rate decreases, allowing detection of renal dysfunction when approximately 40% of nephrons are lost compared to >75% for creatinine. The diagnostic value of SDMA in cats with unilateral renal disease has not been extensively evaluated [8,9].

The significance of pyonephrosis lies in its rapid progression and high risk of systemic complications, including sepsis and acute kidney injury. Since the condition often presents with nonspecific clinical signs, which may delay diagnosis and treatment, early recognition through appropriate diagnostic imaging and prompt intervention to relieve obstruction are critical for improving patient outcomes.

This report describes a 13-year-old cat with massive unilateral pyonephrosis presenting with extreme leucocytosis of 123.22 × 10^3^/µL and elevated SDMA despite normal creatinine concentration. The purpose of this report is to document the clinical, laboratory and imaging findings, emphasise the importance of considering pyonephrosis in the differential diagnosis of large abdominal masses in cats regardless of creatinine concentration and highlight SDMA as a sensitive marker of renal dysfunction in unilateral renal disease.

## 2. Case Presentation

### 2.1. Signalment and History

A 13-year-old female castrated European Shorthair cat weighing 4.0 kg was presented to the clinic with symptoms of lethargy, apathy and progressive abdominal distension reported by the owner. No vomiting, diarrhoea, dysuria, polyuria or polydipsia was observed.

### 2.2. Clinical Findings

On physical examination, the cat presented with a markedly distended abdomen, soft and painless on palpation, pale mucous membranes, CRT < 2 s, no enlarged palpable lymph nodes and a temperature of 38.5 °C.

### 2.3. Laboratory Findings

Urinalysis and urine culture were not performed because the patient was dehydrated, oliguric, and urethral catheterisation was not possible due to the patient’s critical condition.

The complete blood count (CBC) revealed non-regenerative anaemia and marked leukocytosis. Red blood cell (RBC) parameters showed a reduction in red cell mass, with RBC count of 3.49 M/µL, haematocrit (HCT) of 18.6% and haemoglobin (HGB) of 6.0 g/dL. The mean corpuscular volume (MCV) was slightly elevated, while mean corpuscular haemoglobin (MCH) and mean corpuscular haemoglobin concentration (MCHC) were within limits, indicating normochromic to mildly macrocytic morphology. Reticulocytes were absent, suggesting a lack of regenerative response. The total white blood cell count was markedly elevated at 123.22 K/µL, representing severe leukocytosis (Table 1). A blood smear evaluation was not performed due to the unavailability of manual differential at the time of presentation.

Serum biochemistry showed mildly elevated aspartate transaminase (AST), mildly elevated symmetric dimethylarginine (SDMA), slightly decreased potassium, chloride and total protein (TP). The results suggested mild dehydration with minimal variations in the parameters. Creatinine 1.5 mg/dL and BUN 23 mg/dL remained within reference values (Table 2).

### 2.4. Imaging

Abdominal ultrasound revealed a large, poorly marginated, anechoic to hypoechoic mass, approximately 12 × 9 cm, occupying the left cranial and mid abdomen and displacing the stomach, spleen and intestines caudally and to the right. The mass had a thickened, irregular wall ∼4–5 mm. The left renal architecture was not identifiable. The right kidney was of normal size, shape and echogenicity, with a non-dilated renal pelvis measuring < 2 mm. The ureter could not be traced in its entirety. Due to the size of the lesion, image archiving was not possible. Ultrasonographic images were not available for inclusion in this report. However, the ultrasound findings were documented in the medical record and confirmed the diagnosis of pyonephrosis.

**Figure 1:**
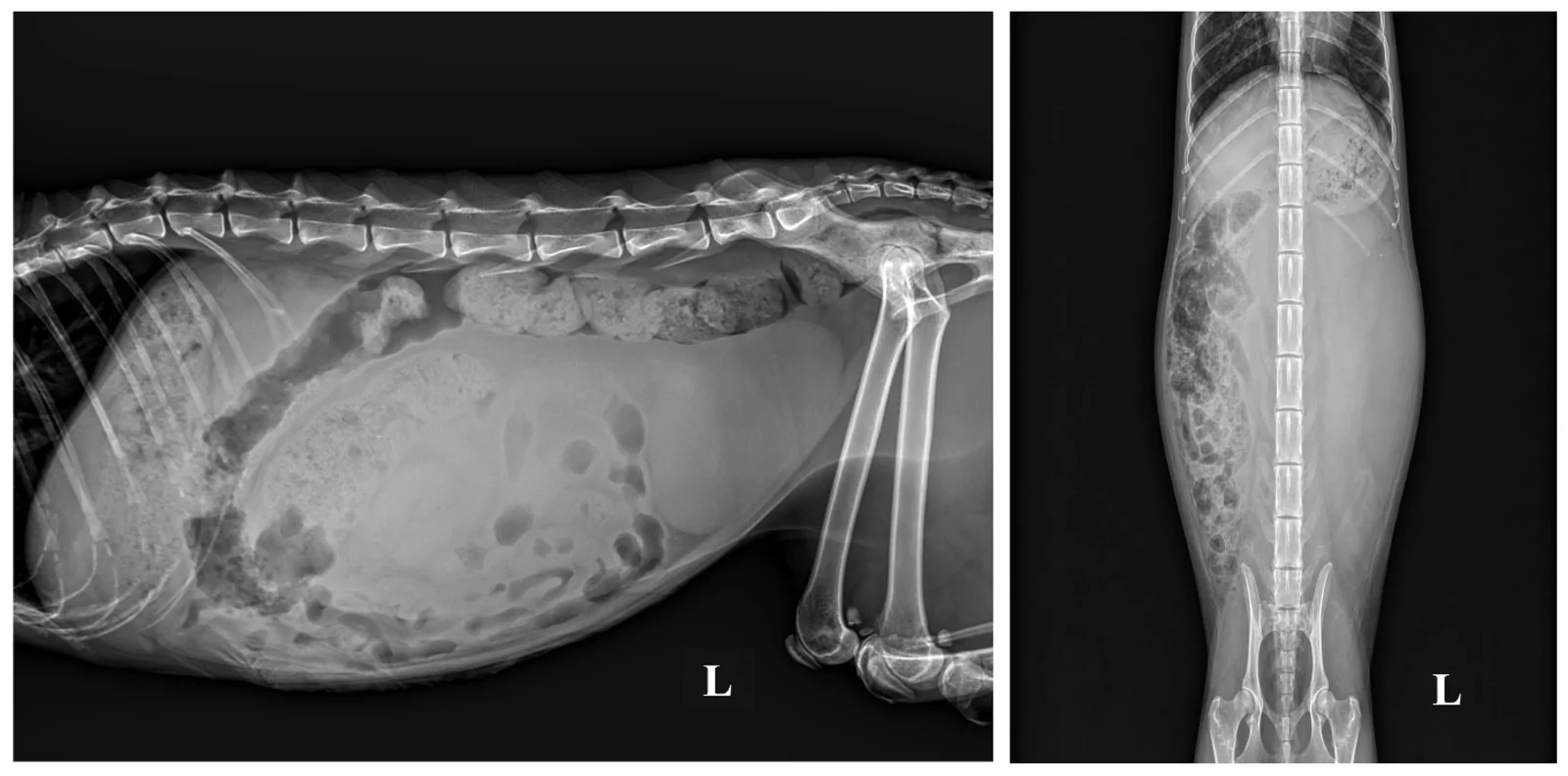
Abdominal radiographs of a patient in right lateral (**Left**) and ventrodorsal projections (**Right**).

Radiographic images were taken in ventrodorsal and right lateral planes. In the right lateral projection, an area of increased opacity was seen in the cranial abdomen. Loops of the small intestines were visibly displaced toward the periphery of the abdominal cavity in the ventral and caudal directions. The large intestine was dilated along its entire length. Gas and a large amount of retained faecal material were visible within the lumen. The ventrodorsal projection shows loops of small intestine displaced towards the right side of the abdominal cavity. The left side of the abdominal cavity revealed a soft tissue opaque mass with indistinct margins. The lesion significantly enlarged the abdominal contour (Figure 1).

### 2.5. Treatment and Outcome

During an ultrasound-assisted abdominal paracentesis, several millilitres of green-coloured purulent mucoid material were aspirated. Microbiological culture of the purulent material yielded *Escherichia coli* susceptible to several antibiotics (Table 3). Culture was performed under aerobic conditions only. Susceptibility was interpreted using the European Committee on Antimicrobial Susceptibility Testing [EUCAST] and Clinical and Laboratory Standards Institute [CLSI] breakpoints. Based on the susceptibility profile, the empirically initiated antimicrobial therapy with amoxicillin–clavulanic acid was continued.

The animal was admitted for hospitalisation and stabilised with intravenous fluid therapy and amoxicillin–clavulanic acid administered at a dose of 10 mg/kg for 24 h (Synulox, Zoetis, Parsippany-Troy Hills, NJ, USA, 140 mg/mL).

Following initial stabilisation, surgical intervention was scheduled for the following day. The patient was premedicated with a mix of dexmedetomidine at a dose of 10 µg/kg (Dexdomitor, Orion Pharma, Espoo, Finland, 0.5 mg/mL) and methadone at a dose of 0.25 mg/kg (Comfortan, Dechra, Northwich, UK, 10 mg/mL). Both drugs were administered intramuscularly. General anaesthesia was induced with midazolam at 0.2 mg/kg (Dormazolam, Dechra, 5 mg/mL) and ketamine at 2 mg/kg (Vetaketam, Vet-Agro, Lublin, Poland, 100 mg/mL), administered intravenously. After endotracheal intubation, anaesthesia was maintained with isoflurane (Isoflurane, Baxter, Deerfield, IL, USA) delivered in a mixture of oxygen and medical air using a rebreathing system, with the fresh gas flow adjusted according to body weight and anaesthetic depth. Perioperative analgesia included robenacoxib at a dose of 2 mg/kg (Onsior, Elanco, Greenfield, IL, USA, 20 mg/mL) administered intravenously. Intraoperative fluid therapy consisted of a balanced crystalloid solution (Ringer’s lactate, Baxter) administered intravenously at 3–5 mL/kg/h, adjusted according to hemodynamic parameters and intraoperative losses.

Intraoperative monitoring included continuous assessment of heart rate (HR), respiratory rate (RR), peripheral oxygen saturation (SpO_2_), end-tidal carbon dioxide (ETCO_2_), and non-invasive blood pressure (NIBP). Body temperature was monitored throughout the procedure and maintained within physiological limits using active warming methods. Anaesthetic depth was assessed regularly based on clinical signs and monitoring parameters and adjusted accordingly.

**Figure 2:**
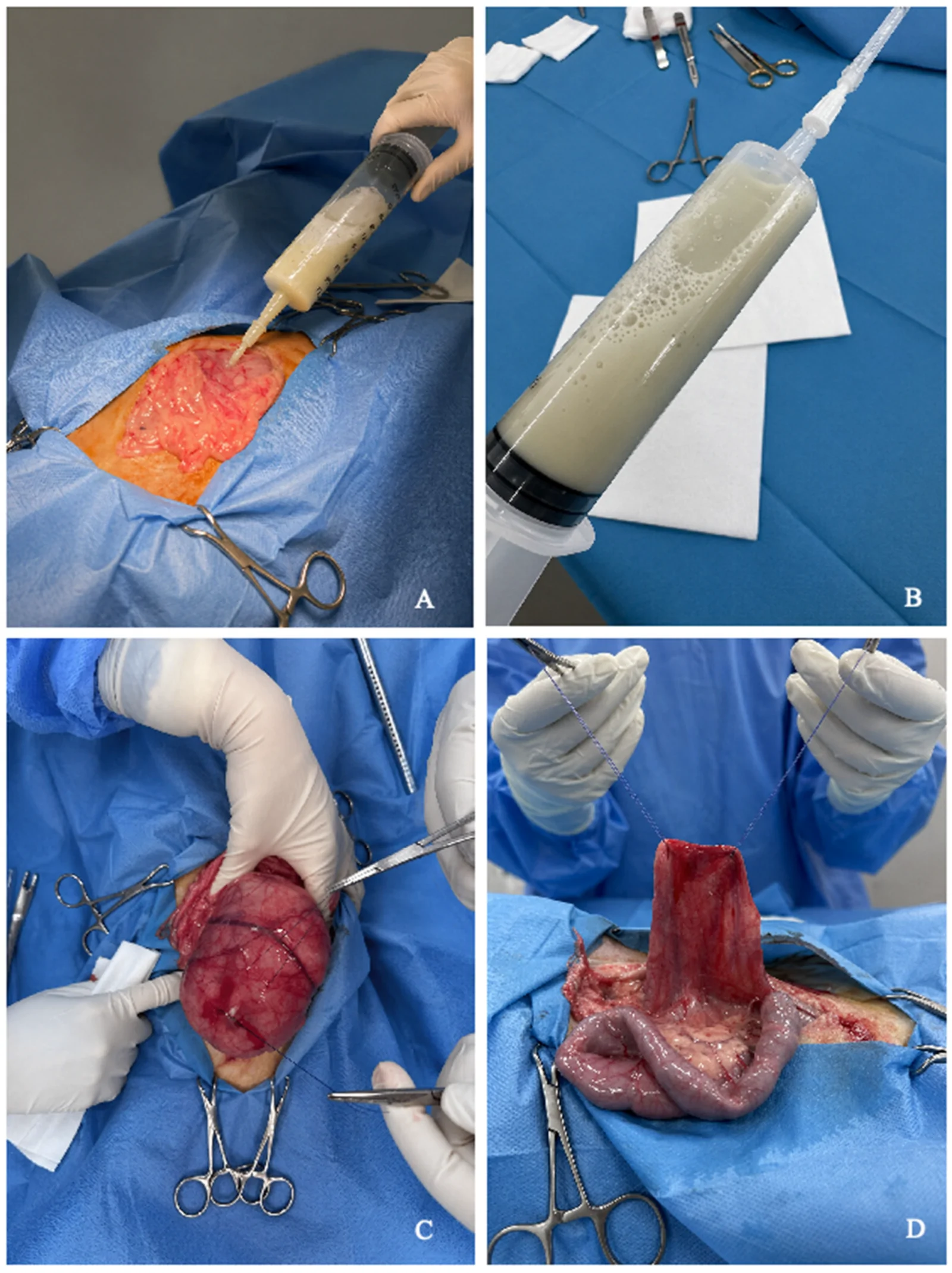
Intraoperative findings. (**A**) Aspiration of purulent content from the abdominal lesion using a sterile syringe immediately after opening the abdominal cavity. (**B**) Syringe containing a large volume of evacuated green purulent exudate. (**C**) Exteriorisation of the cystic lesion from the abdominal cavity, with the renal vein visible on its surface. (**D**) Residual capsule following evacuation of the severe chronic suppurative pyonephrosis with secondary pyelonephritis and ureteritis.

Following induction of general anaesthesia, a ventral midline laparotomy was performed, extending from the xiphoid process to the pubic symphysis along the linea alba. Upon entering the abdominal cavity, a large, fluctuant structure occupying nearly the entire abdominal cavity was immediately identified. To minimise the risk of iatrogenic rupture, partial decompression of the lesion was performed using a sterile syringe and needle (Figure 2). The cystic structure was decompressed by needle aspiration, and approximately 200 mL of green, mucoid, purulent exudate was evacuated and submitted for culture. The volume was estimated by measuring the aspirated syringes during the surgery. Intraoperatively, the abdominal organs appeared markedly displaced and compressed by the mass effect. After initial evacuation of purulent content, the structure was carefully exteriorised from the abdominal cavity to allow detailed assessment of its anatomical relationships. A prominent venous vessel was observed on the surface of the lesion and, upon intraoperative evaluation, was identified as the renal vein, which appeared to have been chronically compressed by the expanding purulent mass. Additionally, a large vessel running from the colon toward the caudal vena cava was noted and appeared markedly distended and compressed. Due to firm adhesions between this vessel and the lesion, complete ligation was required. The liver was enlarged, yellowish, and mottled, exerting pressure on the diaphragm. The gastrointestinal tract, particularly the colon, was distended with a large amount of faecal material. The left kidney was not identifiable, raising suspicion of the renal origin of the lesion. A markedly thickened left ureter was observed coursing toward the mass. The distal ureter was dissected to the level of the ureterovesical junction and ligated proximally. To allow complete excision of the distal ureter and its ureterovesical junction, a small cuff of the urinary bladder wall surrounding the ureteral orifice was resected, resulting in intentional entry into the bladder lumen. The resulting bladder wall defect was closed using a continuous inverting suture pattern without penetration of the bladder mucosa. The distal ureter was dissected to the level of the ureterovesical junction and ligated proximally. To allow complete excision of the distal ureter and its ureterovesical junction, a small cuff of the urinary bladder wall surrounding the ureteral orifice was resected, resulting in intentional entry into the bladder lumen. The resulting bladder wall defect was closed using a continuous inverting suture pattern without penetration of the bladder mucosa. Further evacuation of purulent material was performed. Within the residual capsule, a structure consistent with a severely altered or atrophic kidney remnant was identified. The renal artery and vein were carefully ligated, and the affected tissue was completely excised. Intraoperatively, the liver appeared enlarged, yellow and mottled. This was interpreted as likely reactive hepatopathy or septic hepatopathy secondary to systemic inflammation. Hepatic biopsy was not performed. Post-operatively, liver enzymes and abdominal ultrasound showed improvement, and no specific hepatic therapy was instituted beyond supportive care.

Samples of the purulent exudate were submitted for microbiological analysis, including culture and antimicrobial susceptibility testing. The excised specimen was submitted for histopathological examination to determine the exact nature of the lesion. The abdominal cavity was subsequently inspected, and the abdominal wall was closed in standard layers. All monitored physiological parameters remained within normal limits throughout the procedure.

**Figure 3:**
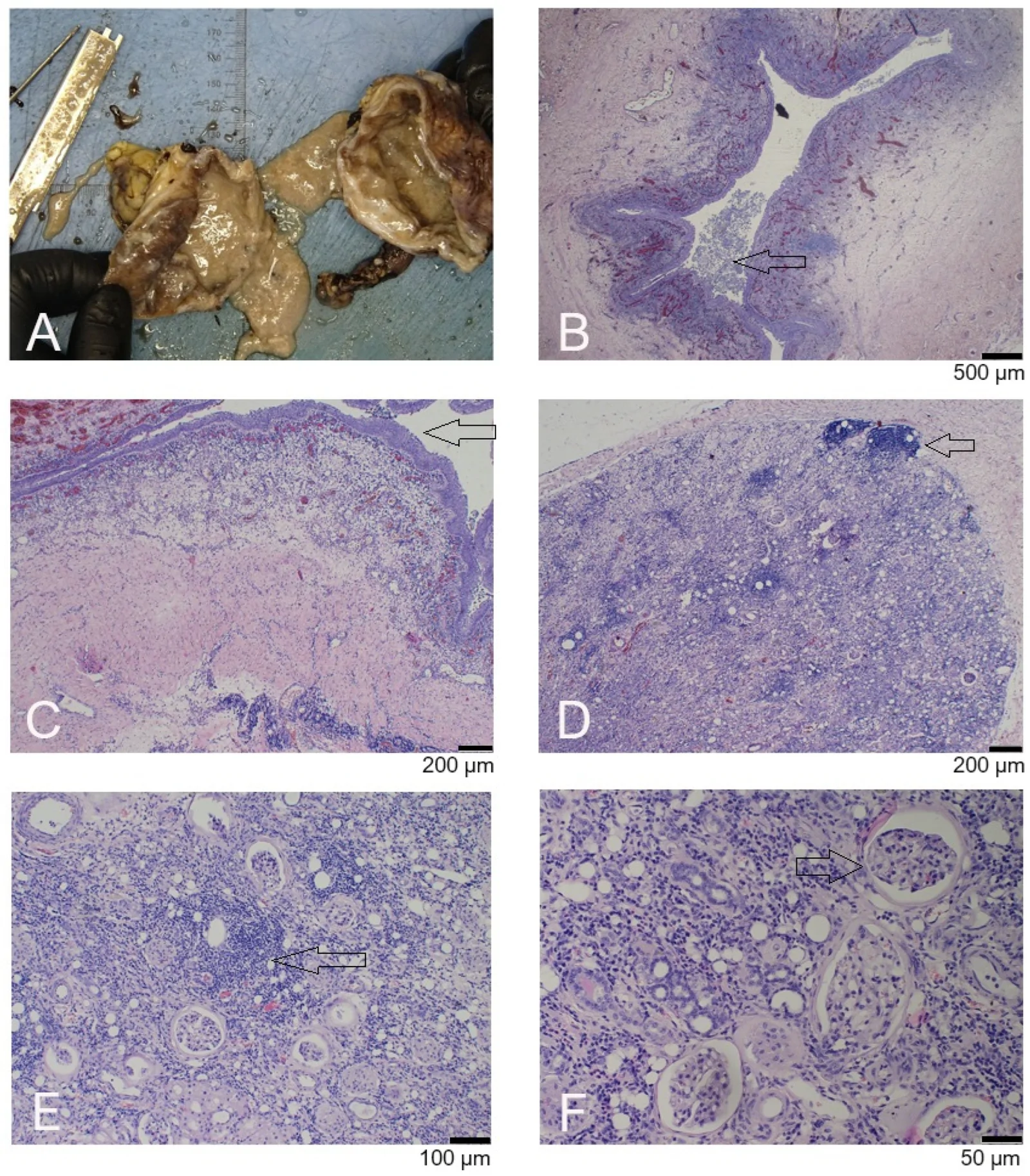
Gross and histopathological findings of the affected kidney and ureter. (**A**) Gross appearance of the excised specimen following nephrectomy, showing severe dilation and deformation of the renal and ureteral structures with thickened walls and abundant purulent content. (**B**) Marked dilation of the renal pelvis with accumulation of purulent exudate (arrow). (**C**) Severe chronic suppurative inflammation with extensive inflammatory cell infiltration, connective tissue proliferation, congestion, and edema (arrow). (**D**,**E**) Renal parenchyma showing multifocal to coalescing inflammatory infiltrates extending into the interstitium (arrows). (**F**) Glomerular changes characterized by fibrosis of Bowman’s capsule and partial glomerular hyalinization (arrows), accompanied by interstitial inflammatory infiltration. No evidence of cellular atypia was identified. Hematoxylin and eosin staining. Scale bars: (**B**) 500 µm; (**C**,**D**) 200 µm; (**E**) 100 µm; (**F**) 50 µm.

Histopathological examination revealed marked dilation of the renal pelvis and ureteral lumen, both filled with purulent exudate. The mucosa exhibited moderate to severe inflammatory infiltrates, predominantly neutrophils, with fewer macrophages, plasma cells, and lymphocytes. These changes were accompanied by the proliferation of well-vascularised, oedematous, and congested connective tissue. The inflammatory process extended into the renal medulla, while the cortical interstitium showed multifocal to coalescing infiltrates of neutrophils, plasma cells, lymphocytes, and fewer macrophages and eosinophils, with concurrent fibrosis. Bowman’s capsules were frequently fibrotic, and some glomeruli exhibited hyalinisation. The ureteral wall was also markedly affected, with dense inflammatory infiltrates and accumulation of purulent material within the lumen. The final diagnosis was severe chronic suppurative pyonephrosis with secondary pyelonephritis and ureteritis extending into the renal interstitium, with concurrent glomerular hyalinisation. No evidence of cellular atypia was identified in the examined tissue sections (Figure 3).

Following surgery, the patient was transferred to a veterinary hospital for continued postoperative care, including supportive treatment, fluid therapy, and close clinical monitoring. Postoperative antimicrobial therapy consisted of amoxicillin–clavulanic acid administered at a dose of 10 mg/kg (Synulox, Zoetis, 140 mg/mL). Analgesia was maintained with robenacoxib at a dose of 2 mg/kg (Onsior, Elanco, 20 mg/mL), with additional rescue analgesia provided using tramadol at a dose of 2 mg/kg (Tralieve, Dechra Veterinary Products, Bladel, The Netherlands, 50 mg/mL) as required. Intravenous fluid therapy was continued postoperatively using a balanced crystalloid solution (Ringer’s lactate, Baxter), administered at a rate of 2–4 mL/kg/h and adjusted based on the patient’s hydration status, urine output, and hemodynamic parameters.

After the procedure, the animal stayed in the hospital for six days due to the high likelihood of a postoperative death and a guarded prognosis. While hospitalised, the patient’s condition improved. The day after the laparotomy, a control ultrasound was performed. Examination revealed a large amount of free fluid in the region of the liver and kidneys, with a lesser amount in the caudal abdominal region. The right kidney appeared normal, with a non-dilated renal pelvis. The liver was of normal size, and its parenchyma exhibited multiple areas of fibrosis. The following day, a repeat ultrasound examination revealed no free fluid in the abdominal cavity. A control blood test was also performed. Postoperative recovery was uneventful. CBC results six days after surgery showed a leukocyte count decreased to 44.81 × 10^3^/µL (Table 4). The cat was discharged with antimicrobial therapy with amoxicillin–clavulanic acid 10 mg/kg PO q12h, which was continued for a total of 10 days after discharge.

At the 3-month follow-up, the cat was clinically healthy, active, with a normal appetite and no abdominal distension.

## 3. Discussion

Pyonephrosis is a severe suppurative infection of the renal collecting system characterised by the accumulation of purulent material [3]. In cats, pyonephrosis is most commonly reported in association with ureteral obstruction, most frequently caused by ureterolithiasis and strictures. However, an underlying obstructive cause is not identified in all cases [1,10,11,12,13]. In the present case, no ureteroliths, ureteral strictures or other obstructive lesions were identified at surgery. The cause of the marked ureteral thickening observed intraoperatively remains undetermined.

The clinical presentation was dominated by lethargy, dehydration, and abdominal distension with marked leukocytosis of 123.22 × 10^3^/µL. This degree of leukocytosis was most consistent with a leukemoid reaction. Leukemoid reactions can occur secondary to severe suppurative inflammation [5,6,7] with emergency granulopoiesis as the main mechanism driving this response [14]. Unfortunately, due to the patient’s critical status, a manual blood smear was not evaluated at presentation; therefore, toxic neutrophils were not assessed. However, in this case, hematopoietic neoplasia was considered less likely. This was based on the rapid decrease in WBC count following surgical drainage of the septic focus. It was also based on the absence of neoplastic features on histopathology. Six days post-operatively, the leukocyte count decreased to 44.81 × 10^3^/µL. This supports a reactive inflammatory process rather than neoplasia because, in neoplasia, leucocytosis would not markedly decrease following removal of the inflammatory focus [6].

Renal parameters were notable for mildly elevated SDMA at 17 µg/dL, with creatinine and BUN remaining within reference values. In this cat, there was a complete loss of function of one kidney while the contralateral kidney appeared grossly normal. The mild SDMA elevation may reflect an early change in global renal function. Several studies have reported that SDMA may be more sensitive than creatinine for detecting decreased GFR in cats [9,15,16]. Moreover, cats can maintain normal creatinine even after unilateral nephrectomy [2,17]. This is due to compensatory hypertrophy of the remaining kidney. This suggests that SDMA may detect renal dysfunction earlier than creatinine in some cats with unilateral renal disease; however, further cases are needed to confirm this observation.

In geriatric cats, the differential diagnosis for a large abdominal mass includes neoplasia (e.g., lymphoma or renal carcinoma), polycystic kidney disease, peritoneal effusion due to FIP, and organomegaly secondary to an abscess [11,12,13]. The marked leucocytosis initially raised suspicion of hematopoietic neoplasia. However, abdominal imaging was diagnostic in this case; ultrasound revealed a large, poorly marginated mass with a thickened wall, replacing the left kidney. No identifiable renal architecture was present, while the contralateral kidney appeared normal. Due to the size of the lesion and the patient’s instability, image archiving was not possible. Surgical exploration confirmed a cystic, thin-walled structure containing approximately 200 mL of green, mucoid, purulent exudate, whose microbiological culture yielded *Escherichia coli*, thereby definitively distinguishing pyonephrosis from neoplasia.

Histopathology confirmed severe suppurative inflammation without cellular atypia, excluding neoplastic transformation. This case highlights that pyonephrosis must be included in the differential diagnosis of large abdominal masses in cats, even in the absence of classic signs such as dysuria or azotaemia.

The treatment of choice for unilateral pyonephrosis is nephrectomy combined with appropriate antibiotic therapy based on culture and susceptibility. Prognosis depends on the function of the contralateral kidney. Cats with unilateral nephrectomy and a normal remaining kidney can maintain long-term normal renal function [2,17]. The patient in this report remained clinically healthy 3 months post-operatively, supporting a favourable prognosis when surgical removal of the septic focus is possible.

## 4. Conclusions

This case report documents massive unilateral pyonephrosis in a geriatric cat associated with extreme leucocytosis consistent with a leukemoid reaction and elevated SDMA despite normal creatinine and BUN concentrations. The rapid marked decrease of leukocyte counts following nephrectomy supports a reactive inflammatory process rather than hematopoietic neoplasia.

The findings of this case emphasise that pyonephrosis should be included in the differential diagnosis of large abdominal masses in cats, even in the absence of azotaemia or urinary tract signs. Moreover, extreme leucocytosis should not automatically prompt evaluation for bone marrow neoplasia when a large septic focus is found, as leukemoid reaction can occur secondary to pyonephrosis. Additionally, this case suggests that SDMA may help detect renal dysfunction earlier than creatinine in some cats with unilateral renal disease and should be measured in geriatric cats with vague clinical signs and abdominal masses. However, further cases are needed to confirm this finding.

Early diagnosis through abdominal imaging and rapid surgical intervention with unilateral nephrectomy can result in a favourable outcome even in geriatric cats with severe systemic inflammatory response. Future reports of feline pyonephrosis should include evaluation of SDMA and serial monitoring of leukocyte counts to further characterise the systemic response to this condition.

## Figures and Tables

**Table 1:** Complete blood count (CBC) results of the patient before diagnosis. H, above the reference range; L, below the reference range.

Item	Result	Reference Range
RBC	3.49 L	6.54–12.2 M/µL
HCT	18.6 L	30.3–52.3%
HGB	6.0 L	9.8–16.2 g/dL
MCV	53.3 H	35.9–53.1 fL
MCH	17.2	11.8–17.3 pg
MCHC	32.3	28.1–35.8 g/dL
RDW	18.9	15–27%
RDW%	18.9	15–27%
RETIC	<reference range	3–50 K/µL
WBC	123.22 H	2.87–17.02 K/µL
NEU%	82.6	
MONO%	3.3	1–4%
EOS%	0.1 L	2–12%
BASO%	0.4	
NEU	101.83 H	2.3–10.29 K/µL
Lymphocytes	16.74 H	0.92–6.88 K/µL
LYM%	13.6 L	20–55%
MONO%	4.09 H	0.05–0.67 K/µL
EOS#	0.12 L	0.17–1.57 K/µL
Basophils	0.44 H	0.01–0.26 K/µL
PLT (Thrombocytes)	226	151–600 K/µL
MPV	17.6	11.4–21.6 fL
PCT	0.4	0.17–0.86%

**Table 2:** Serum biochemistry results of the patient before diagnosis. H, above the reference range; L, below the reference range.

Item	Result	Reference Range
ALKP	<10 L	14–111 U/L
ALT	96	12–130 U/L
Amylase	476 L	500–1500 U/L
AST	97 H	0–48 U/L
BUN	23	16–36 mg/dL
CA	8	7.8–11.3 mg/dL
Cholesterol	76	65–225 mg/dL
Chlorine	109 L	112–129 mmol/L
Creatinine	1.5	0.8–2.4 mg/dL
Glucose	78	71–159 mg/dL
Potassium	3.4 L	3.5–5.8 mmol/L
Lipase	266	100–1400 U/L
Sodium (Na)	156	150–165 mmol/L
PHOS	4.8	3.1–7.5 mg/dL
TBIL	0.4	0–0.9 mg/dL
TP	5.5 L	5.7–8.9 g/dL
BUN/CREA	16	
NA/K	46	
GLOB	3.9	2.8–5.1 g/dL
ALB/GLOB	0.4	
Osm Calc	310	mmol/kg
SDMA	17 H	0–14 µg/dL

**Table 3:** Antimicrobial susceptibility profile of *Escherichia coli* isolated from purulent material. Interpretation based on EUCAST/CLSI criteria.

Category	Antimicrobial Agents
Susceptible (S)	Amoxicillin–clavulanic acid; Enrofloxacin; Florfenicol; Cefuroxime; Cefoperazone; Gentamicin; Ceftriazone; Trimetoprim
Intermediate (I)	Lincomycin-spectinomycin; Ciprofloxacin; Azithromycin; Tobramycin
Resistant (R)	Clindamycin; Ampicillin; Doxycycline; Cefovecin; Ceftazidime; Marbofloxacin; Piperacillin; Tazobactam, Cephalexin, Tylosin

**Table 4:** CBC results of the patient six days after the surgery. ↑, above the reference range; ↓, below the reference range.

Item	Result	Reference Range
RBC	3 ↓	6.54–12.2 M/µL
HCT	17.4 ↓	30.3–52.3%
HGB	5.1 ↓	9.8–16.2 g/dL
MCV	58 ↑	35.9–53.1 fL
MCH	17	11.8–17.3 pg
MCHC	29.3	28.1–35.8 g/dL
RDW	19	15–27%
RDW%	19	15–27%
RETIC	17	3–50 K/µL
WBC	44.81 ↑	2.87–17.02 K/µL
NEU%	87.3	
MONO%	2	1–4%
EOS%	0.7 ↓	2–12%
BASO%	0.1	
NEU	39.09 ↑	2.3–10.29 K/µL
Lymphocytes	4.43	0.92–6.88 K/µL
LYM%	9.9 ↓	20–55%
MONO%	0.91 ↑	0.05–0.67 K/µL
EOS#	0.32	0.17–1.57 K/µL
Basophils	0.06	0.01–0.26 K/µL
PLT (Thrombocytes)	190	151–600 K/µL
MPV	18	11.4–21.6 fL
PCT	0.4	0.17–0.86%

## Data Availability

The original contributions presented in the study are included in the article. Additional anonymized raw clinical and radiographic data are available from the corresponding author upon reasonable request, due to privacy and ethical restrictions related to clinical patient records.
